# Emergence, persistence, and positive selection of yellow fever virus in Colombia

**DOI:** 10.3389/fmicb.2025.1548556

**Published:** 2025-04-07

**Authors:** Lester J. Perez, Laura S. Perez-Restrepo, Karl Ciuoderis, Jaime Usuga, Isabel Moreno, Vanessa Vargas, Angela J. Arévalo-Arbelaez, Michael G. Berg, Gavin A. Cloherty, Juan Pablo Hernández-Ortiz, Jorge E. Osorio

**Affiliations:** ^1^Infectious Diseases Research, Abbott Diagnostics, Chicago, IL, United States; ^2^Abbott Pandemic Defense Coalition, Chicago, IL, United States; ^3^GHI One Health Colombia, Universidad Nacional de Colombia, Medellín, Colombia; ^4^Faculty of Life Sciences, Universidad Nacional de Colombia, Medellín, Colombia; ^5^Department of Pathobiological Sciences, School of Veterinary Medicine, University of Wisconsin, Madison, WI, United States; ^6^Global Health Institute, University of Wisconsin, Madison, WI, United States

**Keywords:** yellow fever virus, acute febrile illness, deimmunization, positive selection, phylodynamic, whole genome sequencing

## Abstract

Yellow fever virus (YFV) is an arbovirus that causes acute febrile illness (AFI), in tropical areas of South America and Africa. Through a 2020–2023 AFI study in Leticia, Colombia, leveraging metagenomic next-generation sequencing (mNGS), we identified and isolated YFV (LET1450). Phylogenetic analysis showed this strain belongs to South American genotype II (SamII), linked to Peruvian and Bolivian sequences emerging around 1989. Phylodynamic analysis indicates these strains, with a unique genetic makeup, could have reduced vaccine susceptibility, and due to positive Darwinian selection have an enhanced adaptive capacity. Antigenic analysis identified additional immune-evasive traits and this strain’s potential for wider Latin American spread. Phylogeographic reconstruction demonstrated the persistence of YFV in Colombia is not due to repeated external introductions, but results from continuous, cryptic internal circulation. This study highlights the crucial role of mNGS in monitoring emerging strains and underscores the need for genomic surveillance of YFV and other arboviral infections.

## Highlights

Despite decades of control efforts, vaccination coverage for Yellow Fever Virus (YFV) is variable and YFV remains a significant cause of acute febrile illness (AFI) in tropical regions of South America and Africa. A potential vaccine-escape strain of YFV belonging to the poorly characterized South American genotype II was identified in a febrile patient by next generation sequencing. The identification of specific adaptive mutations suggests the virus’ capacity to evolve will enable its expansion beyond current geographic boundaries. Recognition of YFV’s continuous circulation in the Amazon region and the emergence of resistance should heighten awareness and inform public health measures.

## 1 Introduction

Yellow fever (YF) is an acute, vector-borne zoonotic illness endemic to tropical areas in America and Africa. This disease is caused by yellow fever virus (YFV) and it is transmitted by several mosquito species within the *Aedes*, *Sabhetes*, and *Haemogogous* genera (Cardoso et al., 2010). YFV belongs to the *Flaviviridae* family that contains other viruses of clinical importance such as dengue virus (DENV), Zika virus (ZIKV), West Nile virus (WNV), and Japanese encephalitis virus (JEV) (Gardner and Ryman, 2010). Most infections with YFV are asymptomatic or present as self-limiting, non-specific febrile illness, but occasionally, patients progress to a more severe disease phase resulting in hepatorenal failure and death (Paules and Fauci, 2017). Yellow fever affects more than 200,000 people per year worldwide, with at least 60,000 deaths annually (Malik et al., 2023). Although there is currently no specific anti-viral drug for YF available, a live attenuated vaccine (17D) has been effectively used since the 1930s to prevent disease (Kieffer et al., 2019).

Sporadic outbreaks of YF have been reported in the southeastern and northeastern areas of Colombia, including the Amazon region which represents about 30% of the landmass (Instituto Nacional de S, 2023). During the past 20 years, at least 215 cases of sylvatic YF cases were confirmed primarily in departments located within or bordering the Amazon region (Vichada, Guaviare, Meta, Caquetá, Vaupés, Amazonas). The most recent case reported occurred in Leticia (Amazonas) in May, 2023 (Instituto Nacional de S, 2023). Vaccination provides effective, long-lasting immunity and has served as the main prevention strategy for YF control in Colombia (Kieffer et al., 2019). However, with the recent COVID-19 pandemic, vaccination programs were abruptly halted (World Health Organization, 2023a). The resulting low herd immunity, along with massive human migration, have impacted the dynamics of virus transmission. Climatic and environmental factors may also be increasing the likelihood of new YFV outbreaks (Kieffer et al., 2019; Instituto Nacional de S, yellow fever virus 2023; Pan American Health Organization, 2023).

Beginning in December 2020, a hospital-based fever surveillance study has been conducted in four regions of Colombia, part of an ongoing virus discovery research program of the Abbott Pandemic Defense Coalition (APDC) (Averhoff et al., 2022). This program aims to better understand the causes of acute febrile syndrome of unknown origin in hot-spot areas where vector-borne diseases are transmitted. Here we describe the detection, isolation, and genetic characterization of YFV from a febrile patient in the Amazon River basin of Colombia.

## 2 Results

**Figure 1:**
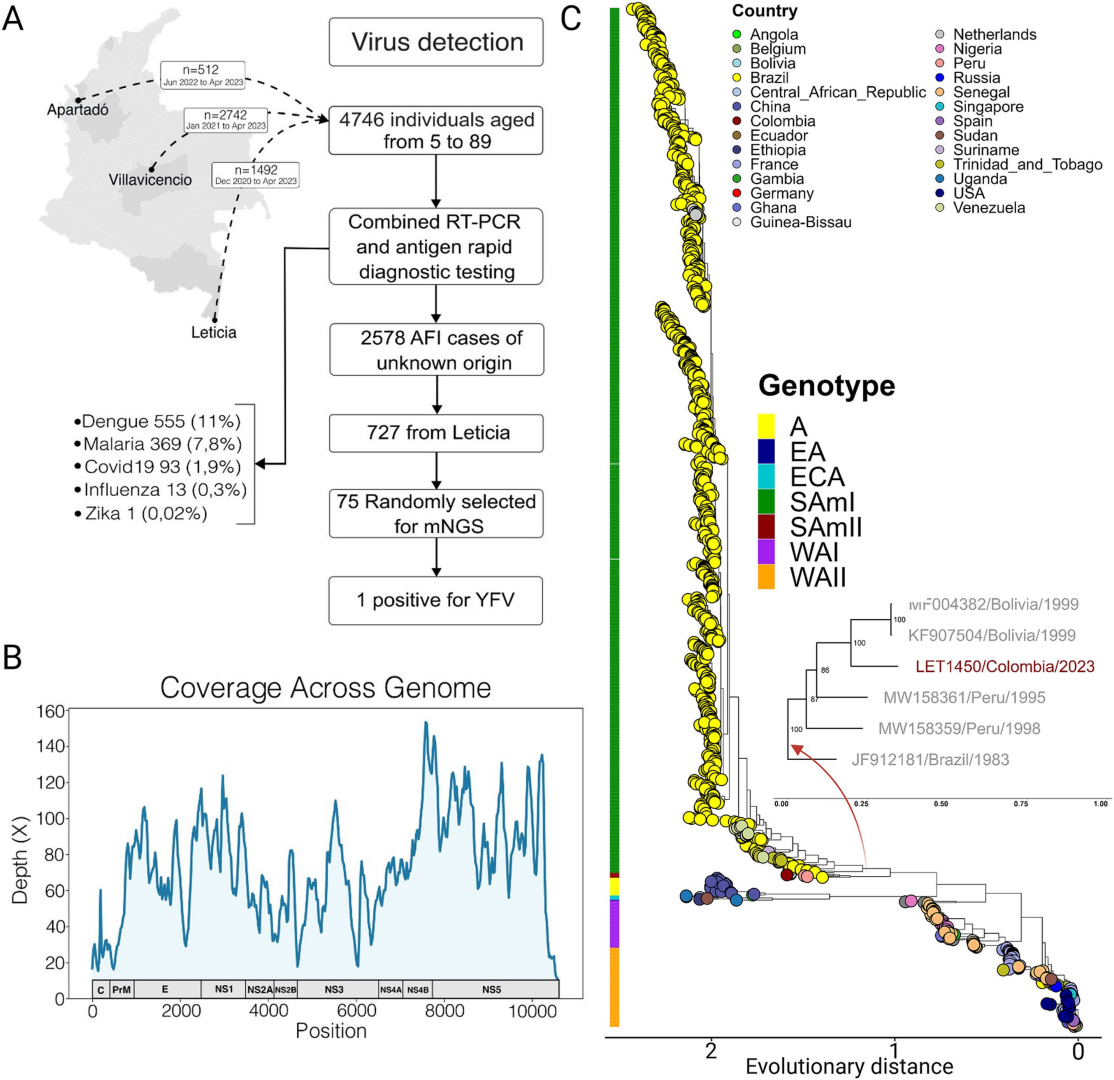
Integrated phylogeny of yellow fever virus. **(A)** Flow chart of the study with the number of samples enrolled, screened, and sequenced prior to the YFV detection. **(B)** mNGS coverage plot across YFV whole genome of patient LET-1450. **(C)** Maximum Likelihood summarized phylogenetic tree constructed from whole-genome sequences of YFV. Strain origins are marked at the tree tips, corresponding to their geographic locations. An external ring classifies the recognized genotypes of YFV, including Asia (A), East African (EA), East Central African (ECA), South American I (SAmI), South American II (SamII), West African I (WAI), and West African II (WAII) genotypes. A focused inset details the SamII genotype, particularly emphasizing the Colombian strain identified in this study. Visualization was created using the *ggtreeExtra* R package.

Acute febrile illness (AFI) surveillance was carried out in four different regions of Colombia between December 2020 and April 2023. Acute-phase sera were collected from 4,746 individuals aged 5 to 96 years, of which 54.3% were females (2,575/4746). Combined RT-PCR and antigen rapid diagnostic testing resulted in detection of dengue (11.7%, 555/4746), malaria (7.8%, 369/4746), COVID-19 (1.9%, 93/4746), Influenza (0.3%, 13/4746), and Zika cases (0.02%, 1/4746). However, after laboratory screening, 53.3% (2,528/4746) of the AFI cases remained of unknown origin (Figure 1A). Of these, 28.8% (727/2528) were from Leticia in the Amazon region. For this study, 7.2% (52/727) of the unknown samples from Leticia were randomly selected for mNGS.

A full genome of YFV was assembled from a 23-year-old indigenous male patient (LET-1450) residing in a semi-urban area of Leticia (Figure 1B). The patient experienced 2 days of fever, vomiting and chills and presented with headache and body/muscle pain. Based on echography, findings indicated enlarged liver, pancreas, spleen, and kidney. Urinalysis and the subject’s additional lab results are described in Supplementary Table S1. A differential diagnosis of leptospirosis was given based on a preliminary diagnosis of urinary tract infection. The subject was treated with cephalosporine, antipyretic, antiemetic, and sodium chloride and was kept overnight for observation. The next day the subject exhibited jaundice and persistent abdominal pain on the left side. Liver function test resulted in increased values as follows: aspartate transaminase 3,260 U/L (reference range 8 to 48 U/L), alanine transaminase 2,280 U/L (reference range 7 to 55 U/L), total bilirubin 2.67 mg/dL (reference range 0.1 to 1.2 mg/dL), and direct bilirubin 2 mg/dL (reference range 0.1 to 0.3 mg/dL). According to the epidemiological survey, the patient indicated prior vaccination against yellow fever virus. Despite these symptoms, the subject refused hospitalization and was lost to follow up.

YFV was successfully isolated following inoculation of infected serum onto Vero cell monolayers. Phylogenetic analysis of all available YFV reference strains delineated seven previously established genotypes (Figure 1C). The LET1450/Colombia/2023 strain clustered with Bolivian sequences, together forming a distinct monophyletic clade within the South American genotype II (SamII), rooted with a Brazilian sequence from 1983 (*zoomed section* Figure 1C). The notably longer branch for the Colombian sequence suggests a prolonged circulation of this strain within Colombia or its vicinity before detection.

**Figure 2:**
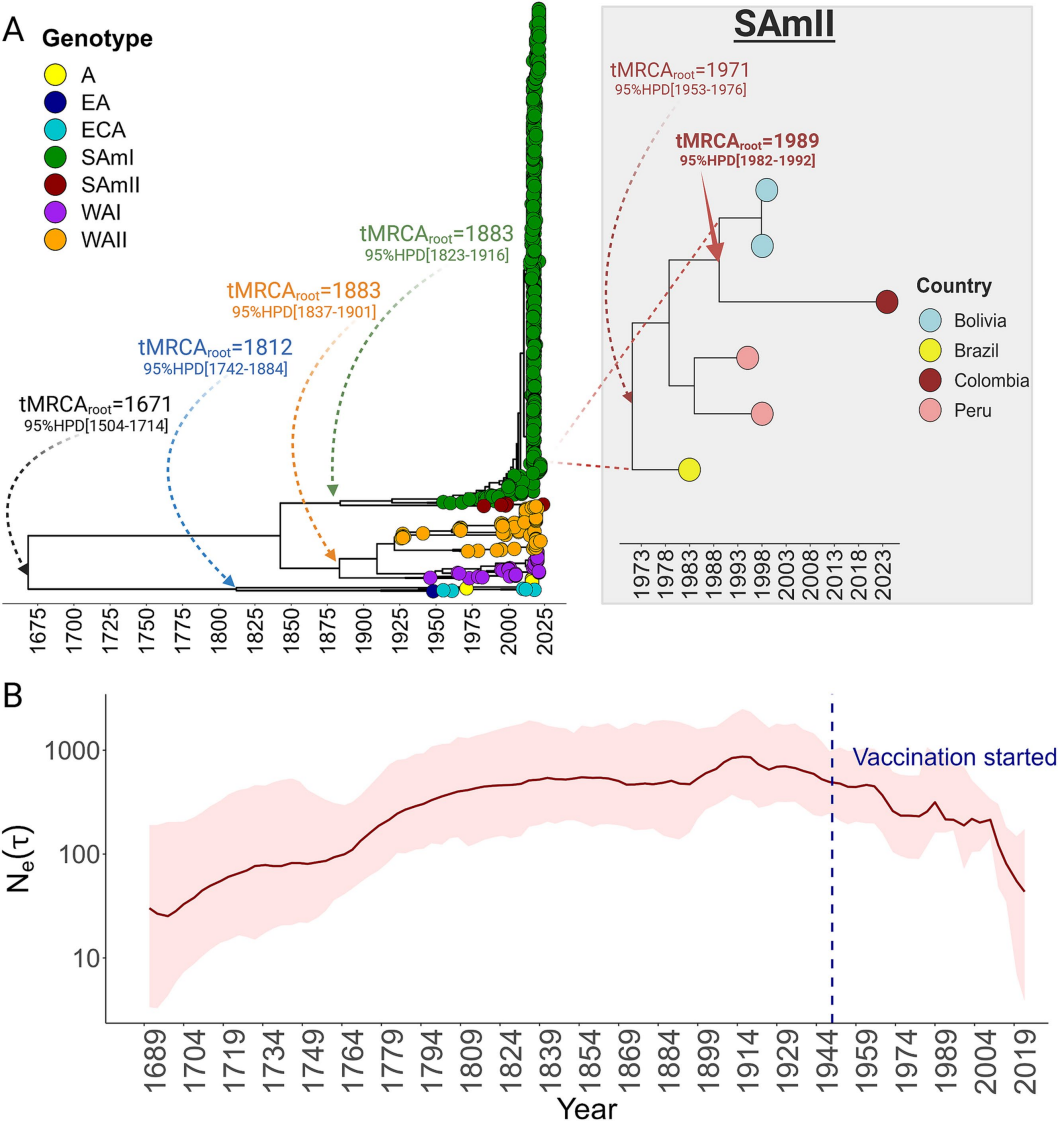
Evolutionary history and demographic reconstruction of yellow fever virus. **(A)** Time-stamped Maximum Clade Credibility (MCC) trees for YFV. The time estimation for the most recent common ancestor (tMRCA) at the root and major diversification events across the phylogeny are also shown. An inset highlights the emergence time of the SamII genotype and the ancestral lineage of the clade encompassing Bolivian and Colombian strains. For all events, the 95% Highest Posterior Density (HPD) intervals are also denoted. **(B)** Demographic history of YFV presented through a Bayesian SkyGrid reconstruction plot. This graph estimates the effective population size (shown as log population size) over generation time (*τ*). A dashed blue line indicates the start of the global vaccination program, and an arrow emphasizes the subsequent increase in the virus population size. This increase coincides with the emergence of the SamII genotype, suggesting a potential correlation between vaccination efforts and genotype evolution.

To determine the evolutionary timeline for the introduction of the SamII YFV lineage into Colombia, we conducted a temporal analysis that traced its emergence and diversification (Figure 2A). We estimated the time to the most recent common ancestor (tMRCA) of YFV overall to be around 1,671 (95% HPD [1504–1714]), aligning closely with the first historically documented case of YF in 1648 (Staples and Monath, 2008). Our analysis further revealed significant evolutionary landmarks, including the diversification of the Asian (A), Eastern Asian (EA), and Central Eastern Asian genotypes around 1812. Additionally, the emergences of the West African (WA), West African II, and South American genotypes were traced back to approximately 1883, and the rise of the South American II lineage, the most recent, emerged around 1971. The ancestor of the Colombian strain was estimated to have arisen around 1989. The Skygrid plot summarizing the entire YFV dataset (Figure 2B) indicates that viral genetic diversity remained stable for over two centuries but experienced a sharp decline coinciding with the global introduction of YFV vaccination efforts that began in ~1950’s (Frierson, 2010). The analysis indeed highlights the vaccine’s effectiveness in reducing transmission and disease. However, we observed a slight uptick in genetic diversity between 1978 and 1985 (Figure 2B). This period corresponds with the further diversification of the South American genotype II (SamII) and the formation of the Peru, Bolivia, and Colombia clusters. These findings suggest that their emergence may have been linked to a positive selection event and/or attributed to an inherent immune evasion characteristic.

**Figure 3:**
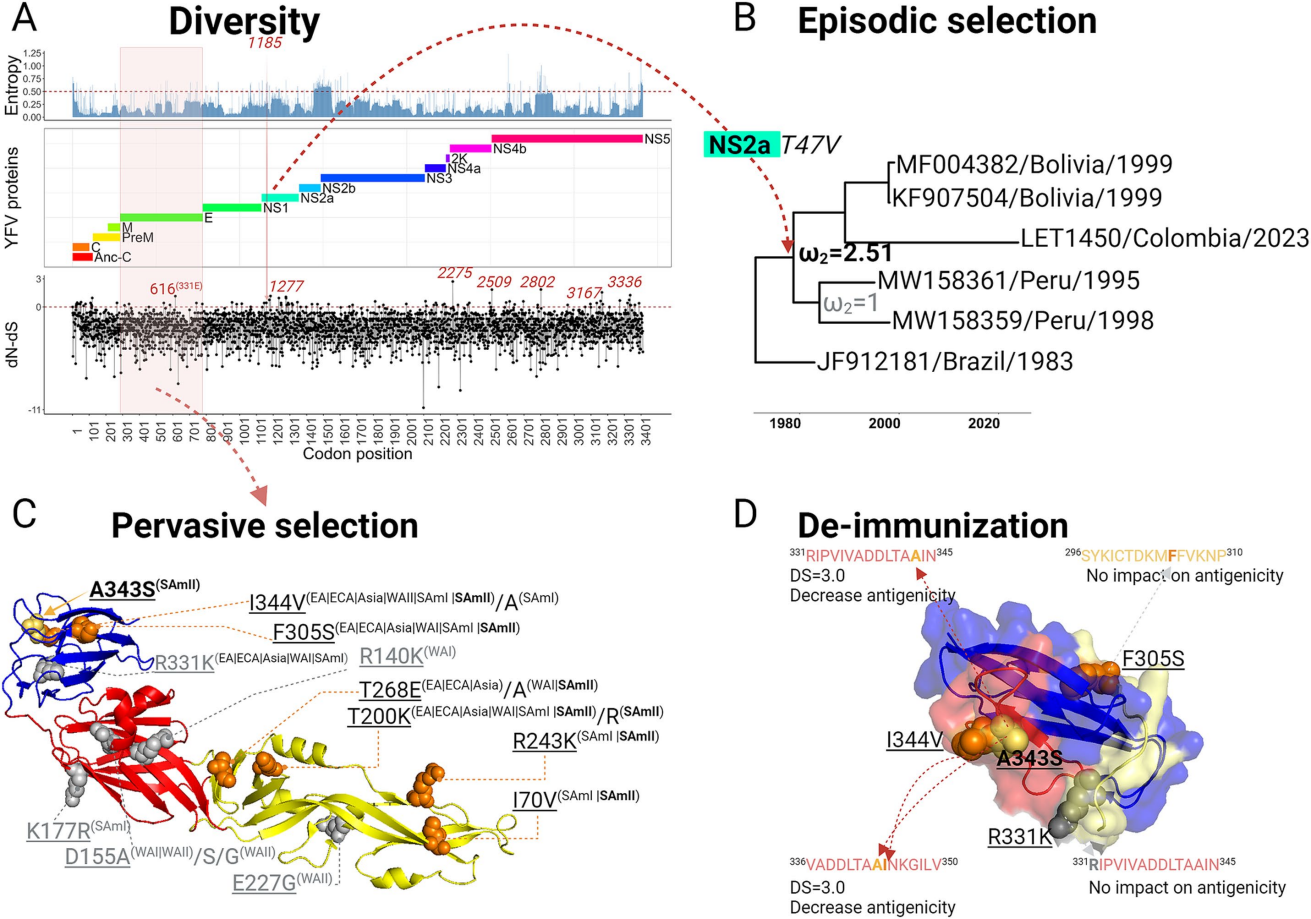
Episodic and pervasive selection as active forces in the evolution of yellow fever virus. **(A)** Analysis of YFV genomic diversification, focusing on the complete coding region. The top panel shows the maps amino acid entropy rates with a cutoff value of 0.5 to underscore highly variable regions, alongside with the map of the premature and mature peptide of YFV along with the disparity between non-synonymous (dN) and synonymous (dS) rates (dN-dS). Codon-specific rates of dN and dS substitutions obtained from the Datamonkey web-server using the SLAC method are plotted. Specific sites (the region in the case of the E protein) identified as positively selected are highlighted in red. **(B)** Episodic selection analysis for the YFV SamII genotype is detailed, where diversification nodes were individually assessed as foreground branches. A node indicating episodic selection is identified by a 𝜔2 value greater than 1 when daughter branches were chosen as foreground. The specific site associated with this selection event is also presented (Supplementary Table S2). **(C)** A 3D representation of the E glycoprotein (PDB ID: 8ofn) shows the domains in distinct colors: Domain I (DI) in red, Domain II (DII) in yellow, and Domain III (DIII) in blue. Positively selected sites resulting from pervasive selection are indicated as spheres (Supplementary Tables S3, S4). Sites with no amino acid replacements in the SamII genotype are in gray, while those with replacements in SamII and other genotypes are in orange. A unique site exclusive to SamII strains is highlighted in gold. Positions are annotated according to the PDB structure numbering, and the corresponding amino acid replacements and genotypes are detailed. **(D)** Analysis of deimmunization effects induced by amino acid replacements at positively selected site exclusive to contemporary SamII strains (marked with red spheres), including the Colombian strain. Deimmunization scores (DS) are noted, alongside the impacts on each antigenic peptide.

The Single Likelihood Ancestor Counting (SLAC) method was deployed to describe the complex landscape of positive nucleotide selection events occurring across the YFV genome (Figure 3A). In parallel, we assessed diversification at the codon level, together with Shannon entropy mapping, to visualize amino acid changes. This combined strategy revealed regions characterized by high levels of diversity and indicative of positive selective pressure, but in general, most of the genome appeared to be under neutral selection or purifying selection (Figure 3A). This finding is consistent with the estimated evolutionary rate of YFV, which is 6.33×10^−4^ (95% HPD interval: 5.3×10^−4^ to 7.1×10^−4^). Sites under positive Darwinian selection are likely to herald the emergence of new viral strains. Hence, a branch-site analysis was performed to gain insight into the evolutionary dynamics giving rise to the SAmII genotype sequences from Peru, Bolivia, and Colombia. Results indicate that this cluster did emerge as a result of positive, episodic selection, with a statistically supported (*p* < 0.01) value for dN/dS = 2.51 (Supplementary Table S2; Figure 3B). From the branch-site analysis, the T1185V mutation, located at position 47 in the non-structural protein 2a (Ns2a), was flagged as instrumental in determining this outcome (Supplementary Table S2; Figure 3B). This same site was also identified as being under positive selection in our SLAC method analysis (Figure 3).

Having established that positive selection was driving the emergence of this cluster within the South American II genotype, we next examined these strains’ potential for immune evasion with a site-specific positive selection analysis of the envelope (E) protein. To increase sensitivity compared to SLAC and minimize false positives, positive selection models were contrasted with neutral selection models, according to Perez et al.(Perez et al., 2022), to determine the action of pervasive selection. A variety of YFV genotypes, sampled over different periods, were included in the analysis (Alignment E protein available at https://github.com/LesterJP/YFV_Colombia). The results highlighted several sites under strong positive selection with omega values (dN/dS) > 14, confirmed by significant statistical support (*p* < 0.01) (Supplementary Tables S3, S4). Notably, 7 of the 12 positively selected sites in E were present in the SamII lineage (Supplementary Table S2; Figure 3C), with A343S found exclusively in the Peru/Bolivia/Colombia cluster. This mutation is situated in one of the exposed loops of domain III of the envelope protein responsible for receptor-mediated attachment (Figure 3C). To explore whether the positively selected sites identified in DIII of the envelope protein confer immune evasion capability, particularly to the vaccine, we assessed their de-immunization potential using the approach previously described by Coronado et al. (2019). A de-immunization score (DS) of 3 indicates that the A343S replacement strongly contributes to a reduction in antigenicity (Supplementary Table S1; Figure 3D), as does the adjacent I344V mutation (DS = 3.0), a combination that potentially produces a synergistic effect. Neither the F305S replacement identified on the opposite face of the protein, nor the R3331K (absent in the SamII genotype) located at the proximal loop region, impact the antigenicity of their respective peptides (Figure 3D). While the identification of the A343S suggests this and other mutations may impart unique adaptive features in these regional variants, it will require *in vitro* neutralization assays to determine their impact on vaccine escaping capacity.

**Figure 4:**
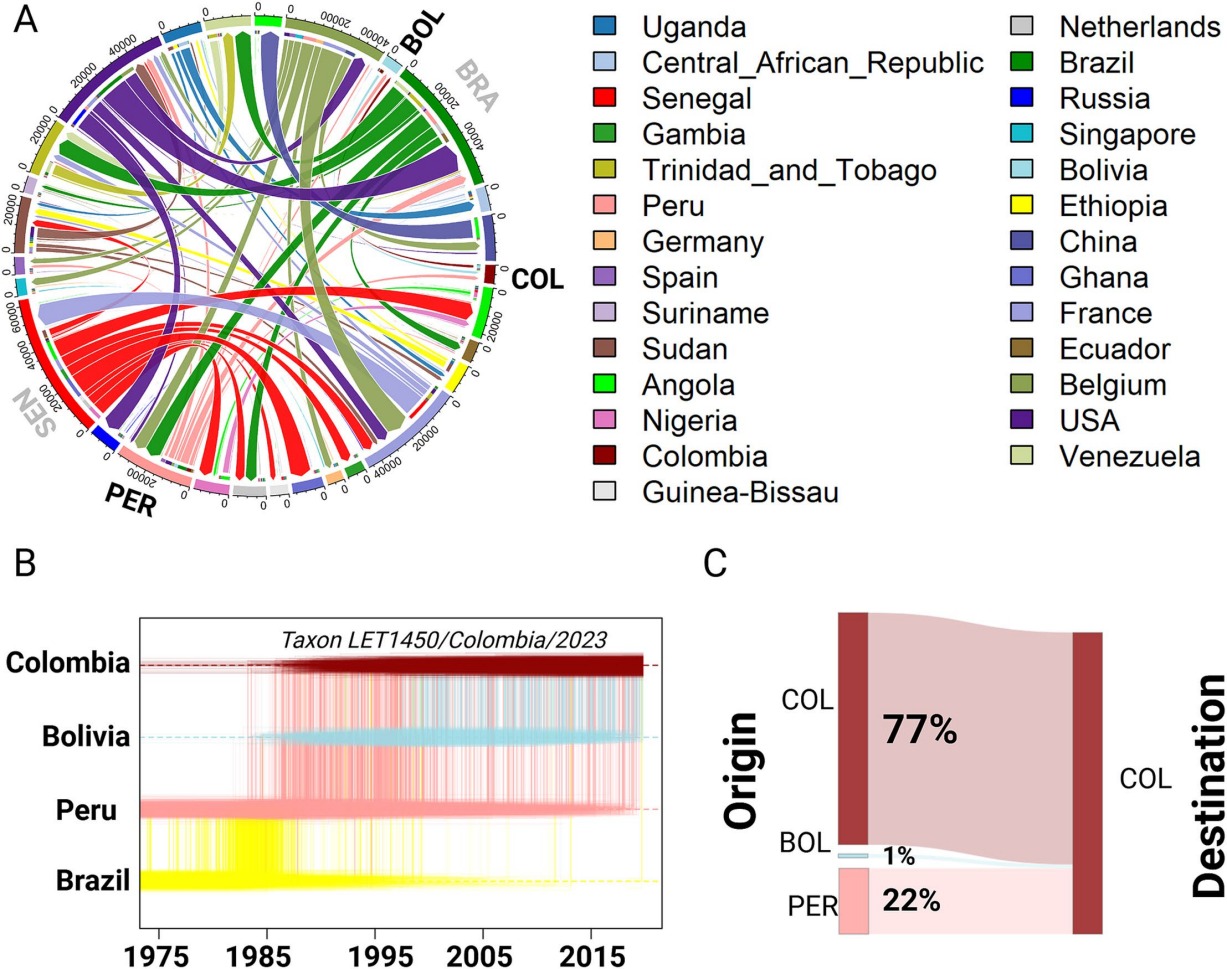
Geographic trajectories of yellow fever virus inferred from discrete phylogeographic analyses. **(A)** Circular migration flow plot that represents the origin and destination of international YFV spreading events. The thickness of each link reflects the estimated frequency of viral movements, determined through *post hoc* analyses of summarized posterior expectations of Markov jump events from the entire history reconstruction. Migration flows are indicated starting from the outer ring and pointing toward their destination, marked by arrowheads. **(B)** The Markov jump trajectory plot details the ancestral transition history for the most ancient genome of the SamII genotype in Colombia, specifically focusing on the strain identified in our current study (taxon name denoted). This plot summarizes trajectories derived from a posterior tree distribution annotated with Markov jump history, utilizing sampling location and Bayesian Stochastic Search Variable Selection (BSSV) model. Horizontal lines represent periods where a specific location state is maintained in the virus spatiotemporal ancestry, while vertical lines indicate Markov jumps between locations. **(C)** Sankey plot showing the migration flow, summarizing the persistence of a particular lineage identified in Colombia among countries where the virus circulates. The width and percentage of each flow represent the formation of unique lineages that originated from the same geographically specific country, considered over the evaluation period. This is weighed against the total number of unique lineages formed. For clarity, the origin and destination countries are denoted using their ISO three-letter codes.

To determine the origins and global dissemination of YFV, we performed a discrete phylogeographic analysis. The topological structure produced from the Markov jump count plot was consistent with the genotypic evolution of the virus, tracing the root to Uganda and highlighting the role of Brazil and Senegal in its worldwide dispersion (Figure 4A; Supplementary Figure S1). Regarding Colombia, our analysis pinpointed two major importation events from Peru and Bolivia, with transmission back to Bolivia (Figure 4A). To determine the most likely origin of the initial introduction of the SamII genotype into Colombia, we employed the *TaxaMarkovJump* history reconstruction method (Lemey et al., 2021). This approach pointed to a ‘jump’ from Brazil to Peru, identifying Peru as the primary source of simultaneous introductions into Colombia and Bolivia (Figure 4B). Furthermore, the Markov jumps, as represented in Figure 4B by vertical lines, were originated from Peru and Bolivia and leading into Colombia, which indicates multiple YFV introductions from these neighbors.

To address the key question of whether the prevalence of YFV strains in Colombia results from repeated external introductions or from continuous and cryptic endemic circulation, we conducted a *post hoc* analysis derived from the entire reconstructed history of the Markov jump trees. The *PersistenceSummarizer* tool (Lemey et al., 2021) allowed us to examine the flow of viral strains within a unique lineage that originated from the same country. Our study period spanned from 1975, the ancestral time point (*T_a_*), to 2023, the time of evaluation (*T_e_*), and revealed that circulation dynamics within Colombia were largely driven internally. Specifically, the analysis showed that 77% of the virus circulating in Colombia originated from within Colombia itself. Only 22% traced back to Peru and a mere 1% was linked to Bolivia (Figure 4C). These findings demonstrate that the majority of YFV activity in Colombia is due to endemic persistence rather than external introductions.

## 3 Discussion

Acute Febrile Illness (AFI) is caused by a wide variety of pathogens but presents with high similar, non-specific symptoms. Clinicians commonly refer to a AFI as a fever of unknown origin without an initially obvious etiology or without localizing signs (Ryan, 2024). It is a major reason for seeking healthcare in low- and middle-income countries in the tropics (Feikin et al., 2011) and its diagnosis is one of the most challenging to make in clinical settings (Peeling and Fongwen, 2022). Malaria and dengue are the most common infections associated with AFI in the tropics (Wangdi et al., 2019). Various geographically restricted pathogens (e.g., Mayaro), as well as the globally prevalent causal agents of AFI (e.g., Chikungunya), also drive the epidemiology of fever across tropical regions (Prasad et al., 2015). However, a high proportion of AFI are inconclusive after routine diagnostic testing and therefore the relative importance of undetected pathogens causing AFI remains under-appreciated (Bottieau and Yansouni, 2018). For example, the burden of AFI caused by arboviruses such as Oropouche virus may be highly underestimated in some regions (Ciuoderis et al., 2022; Moreira et al., 2018). In this study, the subject suffering from YF had been given a diagnosis of suspected leptospirosis during the early stages of the disease. Thus, considering the barriers to accessing targeted laboratory testing in resource-limited healthcare settings, such as those in the Amazon region of Colombia, the early confirmation of YFV infection was indeed challenging. Molecular tools, such as metagenomic sequencing, may help to fill this gap. Several research studies have demonstrated the utility of mNGS for pathogen discovery and surveillance of neglected diseases (Chen et al., 2023; Wright et al., 2022; Jerome et al., 2019). In this context, the Abbott Pandemic Defense Coalition (Averhoff et al., 2022) is a key initiative which is helping to reduce this gap in information, while also providing with local capacity building, resources and training for enhancing the detection of uncommon and novel pathogens causing AFI worldwide.

Since 1970, YF has re-emerged as a public health threat in several tropical regions of the world. The largest outbreak of the past five decades in the Americas took place in Brazil between 2016 and 2018, with transmission outside the Amazon region generating hundreds of deaths (de Oliveira et al., 2020). Recently, persistent YFV circulation and outbreaks were reported in Africa at locations with little or no underlying immunity or in areas with no history of YF vaccination (World Health Organization, 2023b). In Colombia, two sylvatic YF cases were confirmed during 2023, occurring in the Amazon region, out of the 44 suspect cases investigated in the country (Instituto Nacional de S, 2023). In our globally interconnected world, marked by extensive international travel and trade, awareness of YFV re-emergence in regions with no prior exposure or immunity is more pressing than ever before and highlights the challenge for global public health (de Oliveira et al., 2020).

The recent emergence of the South American II genotype and the Peruvian/Bolivian/Colombian lineage demonstrates the continuous evolution and regional diversification of YFV in the Americas. The demographic reconstruction, as depicted in the Skygrid plot, illustrated the decline in viral genetic diversity following the commencement of vaccination efforts. Amid the broader scientific controversy around the YFV vaccine, our study agreed with others and underscored its pivotal role in controlling YFV (Rosenstein et al., 2021; Juan-Giner and Hombach, 2024). The slight increase observed from 1978 to 1985, coinciding with the diversification of the SamII lineage, typifies the challenge of uniformly suppressing highly genetically variable lineages through vaccination (Rosenstein et al., 2021). The unique immune responses elicited by these diverse strains can lead to disparate vaccine efficacy across lineages, as evidenced by the breakthrough cases documented by Faria, *et al* in the recent YFV outbreak in Brazil (Faria et al., 2018). Seven individuals infected with the circulating Sam I genotype were previously vaccinated against YFV (Faria et al., 2018). It is important to note that the subject in this study reported being vaccinated against YFV, which suggests the possibility of a breakthrough mutant capable of evading vaccine-induced immunity.

Our temporal analysis, while focused on the SamII genotype in Colombia, also uncovered key events in the timeline of YFV evolutionary dynamics and global dispersal. Our estimated time to the most recent common ancestor (tMRCA) of YFV around 1,671 to the historical documentation of the first yellow fever case in 1648 not only affirms the reliability of the Bayesian analytical methods but reinforces the need for extensive datasets to anchor phylogenetic dating. The diversification of various Asian and African genotypes aligns with periods of increased global mobility, especially during the 19th century when major advancements in maritime technology enhanced oceanic travel and facilitated extensive human movement (Rodrigue, 2020). These developments, along with the growth of international trade and colonial expansion, played a pivotal role in human activity, significantly contributing to viral spread (Galbraith and Barrett, 2009). Ours is the first study to document this historical progression and illustrates that, selective pressures (e.g., vaccines) are still being exerted to which YFV adapts.

The identification of evolutionary forces at work is crucial to understanding the adaptive mechanisms deployed by emerging viral strains (Yu and Cheng, 2022). Our analysis indicated that while most of the YFV genome appears to be under neutral or purifying selection (Figure 3), there were certain regions exhibiting high diversity and subject to positive selection. Focusing on the Peru, Bolivia, and Colombia cluster, we observed positive episodic selection indicative of ongoing region-specific evolutionary adaptation, potentially attributed to mutations such as T1185V in the non-structural protein 2a and A343S in domain III of the envelope protein. The A343S mutation found exclusively in the Peru/Bolivia/Colombia cluster within the SamII genotype provides a unique adaptive feature that likely contributes to immune escape. Indeed, our de-immunization analysis revealed a notable reduction in antigenicity linked to this mutation. Moreover, the emergence of the I344V mutation also found in strains of the SamI genotype adds another layer of complexity, especially as it runs in parallel to an unprecedented outbreak in the extra-Amazonian regions which has surpassed the scale of the last 7 years (Giovanetti et al., 2022). These particular mutations highlight a significant evolutionary step in YFV, potentially impacting both the viral transmissibility and the efficacy of existing vaccines. Vaccine coverage has held steady around 80% but has dropped in recent years during the COVID-19 pandemic (Shet et al., 2022). This lapse provides another window of opportunity for YFV to adapt to decreased pressure.

The predominance of internally driven YFV circulation in Colombia (Figure 4), as revealed in our study, challenges the prevailing assumption of the virus’ propagation mainly through external introductions. This shift in understanding calls for a reevaluation of current public health strategies, emphasizing the need for targeted interventions addressing endemic transmission within the country, as recently described in Giovanetti et al. (2023). Considering the complexity of viral dynamics in endemic regions, continuous, comprehensive surveillance systems coupled with reliable diagnostic methods are required to monitor the evolution and spread of YFV within national borders. Given the potential implications for vaccine strategy and virus control, further studies are needed, including *in vitro* cross-neutralization assays, as previously mentioned. Another potential limitation of our study, despite utilizing one of the largest datasets of YFV genomes to date in comparison with other studies (Faria et al., 2018; Giovanetti et al., 2023; Li, 2022), is the limited number of genomes available, specifically for the SamII genotype. This comparatively smaller dataset for SamII genotype may not fully capture the complete genetic diversity and evolutionary dynamics of this genotype, which again underscores the need for more surveillance and sequencing. While this manuscript was under review, a surge in yellow fever cases with a fatality rate of nearly 50% was reported in Colombia and Brazil. According to updates from the Pan American Health Organization (PAHO) and the World Health Organization (WHO), an outbreak in Tolima’s mountainous region has, as of February 2025, led to at least 31 reported cases and 15 deaths. This alarming development underscores the continued public health threat posed by arboviral diseases and highlights the urgent need for coordinated surveillance and intervention efforts, like the APDC (Averhoff et al., 2022) and other networks seeking to detect emerging threats and respond with needed diagnostics is a step in this direction.

## 4 Materials and methods

### 4.1 Ethics statement

This research was conducted in accordance with the principles outlined in the Declaration of Helsinki. All procedures involving human subjects were approved by the Ethics Committee of the Corporación Investigaciones Biológicas (CIB 10102022). Informed consent was obtained from all participants prior to their inclusion in the study. Procedures for Yellow Fever Virus isolation were conducted in the Biosafety Level 3 facility at the University of Wisconsin-Madison under the approved Biosafety protocol # B00000089. Any potential conflicts of interest have been disclosed and managed appropriately.

### 4.2 Patient cohort

A hospital-based fever surveillance study was carried out in four different regions of Colombia (Villavicencio, Acacias, Apartadó and Leticia), beginning in December 2020. Written informed consent was obtained from adults (18 years or older) and informed assent was obtained for children and adolescents (aged 17 years or below). Parent/guardian of minors (<18 years) also provided written consent on their behalf.

### 4.3 AFI case definitions, specimen collections and pre-screening

AFI was defined as a recent onset of fever (body temperature ≥ 38.5°C at the time of consultation or self-reported history within the preceding 7 days) in the absence of an obvious focus of infection. In addition, definition of AFI also included association with non-specific symptoms such as headache, body rash, and muscle and joint pains. Venous blood samples were tested at the point-of-care using rapid diagnostic tests for dengue (SD Bioline Dengue Duo, Abbott) and malaria (SD Bioline Malaria Ag P.f/Pan, Abbott). Serum/plasma samples were stored at −80°C until transportation to a central lab (One Health Genomic Lab (Universidad Nacional de Colombia, Medellin) for further testing. Samples were tested at the central lab by molecular assays (reverse transcription RT-PCR and/or PCR) for detection of malaria, dengue (DENV), Zika (ZIKV), chikungunya (CHIKV), influenza (IV), and severe-acute-respiratory-syndrome-related coronavirus (SARS-CoV-2). Testing was conducted following protocols described elsewhere (Kamau et al., 2013; Waggoner et al., 2016; de-Paris et al., 2012; Corman et al., 2020). This work was reviewed and approved by the Ethics Committee of the Corporación Investigaciones Biológicas (CIB 10102022).

### 4.4 Virus isolation

The infected serum sample (50 μL) was inoculated onto sub-confluent Vero cell monolayers (T-25 flask) and incubated at 37°C with 5% CO_2_ following the protocol previously described (Furtado et al., 2022). Cells were observed daily for cytopathic effects (CPE). After CPE were observed, cell culture supernatant was collected and evaluated for YFV by RT-PCR as described (Nunes et al., 2011). Molecular testing was conducted using the MiniAmp Plus thermal cycler (Applied Biosystems). Virus isolation was conducted in a Biosafety Level 3 facility at the University of Wisconsin-Madison (Biosafety protocol # B00000089).

### 4.5 Metagenomic next generation sequencing (mNGS)

Random-primed cDNA libraries derived from the serum specimens were prepared for mNGS following the protocol described elsewhere (Berg et al., 2020). Briefly, total nucleic acid was converted to cDNA by priming with random hexamers and oligo(dT) using Superscript III (SSRTIII) 1st Strand reagents (Life Technologies, Carlsbad, CA, USA), followed by 2nd strand synthesis with Sequenase V2.0 T7 DNA pol (Affymetrix, Santa Clara, CA, USA). The Nextera XT (Illumina, San Diego, CA) kit was used to create barcoded, metagenomic NGS libraries which were then purified with AxyPrep magnetic beads, quantified on both a Tape Station (Agilent Technologies, Santa Clara, CA) and Qubit fluorometer (ThermoFisher Scientific, Waltham, MA), pooled and diluted to 650 pM for loading. The NextSeq2000 (Illumina, California, USA) platform was used for sequencing and data were analyzed using an open-source cloud-based tool (Kalantar et al., 2020).

### 4.6 Genomic data collection, alignment and phylogenetic analysis

We retrieved all sequence of YFV available in Nextstrain repository^1^ on December 13th, 2023. All downloaded sequences, were aligned together with the sequences obtained in the current study using the MAFFT software v7.453 (Rozewicki et al., 2019) with settings for a “localpair” option alignment. The obtained alignments were then utilized for maximum likelihood (ML) phylogenetic inference using IQ-TREE2 (Nguyen et al., 2015) as described in Perez et al.(Perez et al., 2022). Additionally, two different traits including genotypes, geographic location were mapped into the phylogeny using the ggtree R package (Yu et al., 2017).

### 4.7 Temporal and demographic Bayesian inference

To estimate the temporal emergence and evolutionary rate of YFV, we generated time-scaled phylogenies using BEAST 1.10.5 (Suchard et al., 2018), incorporating the BEAGLE 3 library (Ayres et al., 2019) to improve computational efficiency. The molecular clock parameters employed in our study were based on the framework established by Perez et al. (2023). For our phylogenetic analysis, we selected the SkyGrid tree prior model (Hill and Baele, 2019), which is particularly fitted for handling fluctuating population dynamics over time. Our analytical process involved 16 independent runs conducted on an AZURE cloud server. Each run sampled from Markov Chain Monte Carlo (MCMC) chains at intervals of 9×10^8^ generations, with a sampling frequency of every 9×10^5^ generations. The convergence of these runs was carefully evaluated based on the effective sample size (ESS) of parameter estimates. We keep to the standardized threshold of ESS > 200 for reliable convergence, determined using Tracer 1.7 software (Rambaut et al., 2018). After a 10% burn-in, the runs were combined using the LogCombiner software (Drummond and Rambaut, 2007). For the phylogenetic representation, we constructed a Maximum Clade Credibility (MCC) tree using TreeAnnotator software. Additionally, we employed Tracer 1.7 software (Rambaut et al., 2018) for the SkyGrid reconstruction, which provided a detailed depiction of the demographic history of YFV.

### 4.8 Discrete phylogeographic analysis

To investigate the geographical spread of YFV and identify the origins of external introductions as well as the local emergence of strains in Colombia, we employed discrete trait phylogeographic inference (Lemey et al., 2020) as conducted in Perez et al. (2022). This method allows for a detailed analysis of the geographical distribution and migration patterns of the virus. For further insights, *post hoc* analyses were conducted to estimate MarkovJump transitions and evaluate the persistence of the YFV strain within Colombia. These analyses utilized advanced tools from the BEAST codebase, specifically the TaxaMarkovJumpHistoryAnalyzer, TreeMarkovJumpHistoryAnalyzer, and PersistenceSummarizer developed by Lemey et al. (2020), and available at BEAST GitHub Repository. Visualization of these complex data sets was achieved through a combination of specialized R packages, including MarkovJumpR and circlize and a custom Python scripts.

### 4.9 Positive selection analyses

To thoroughly investigate the influence of pervasive and episodic selection on the evolution of YFV strains, we adapted the methodology described elsewhere (Alfonso-Morales et al., 2015) and incorporated additional modifications as proposed by Orf et al. (2023). Our analysis was structured into three distinct phases. In the first phase, we assessed the genetic diversity of YFV. This involved analyzing the Shannon Entropy and evaluating positive selection across a broad dataset using the Single Likelihood Ancestor Counting (SLAC) (DataMonkey web application) (Weaver et al., 2018) method on the complete coding region of all 1,211 YFV strains in our dataset. Following this, we proceeded to a more focused analysis that included the analysis of episodic selection, specifically targeting the SamII strain of YFV. We utilized the time-stamped MCC tree derived from our temporal analysis of these strains as inputs for the CODEML program within the PAML v.4.9 software package (Yang, 2007). In this analysis we aimed to identify which of both lineages were under positive selection within the complete coding genome of the SamII strains. We conducted branch-site tests on predetermined branches, (foreground branches) and compared them against the background branches. The comparison utilized the alternative Model A and the null Model A1. For the statistic support of this analysis we performed branch-site Likelihood Ratio Tests (LRTs). The significance of the LRTs was determined by the chi-squared (𝜒2) distribution of twice the log ML difference between the two models, with degrees of freedom corresponding to the difference in the number of parameters between the models. Detailed results of this analysis, including the statistical significance of episodic selection events, are presented in Supplementary Table S2. For the assessment of pervasive selection on the envelope protein of YFV, we carefully selected sequences from various genotypes, ensuring each had high-quality annotations of this region. These sequences were chosen based on their representation of different temporal epochs identified in our temporal analysis. From this refined dataset (Supplementary Figure S1), we constructed a new ML tree using the IQ-TREE software. This tree was then utilized as the input for the CODEML program within the PAML v.4.9 software suite, where we applied site models M2 and M8. To accurately identify sites under positive selection, we implemented the Bayes Empirical Bayes method, which calculates posterior probabilities for these sites. To minimize the likelihood of false positives, we conducted a comparative analysis between the models used for detecting positive selection pressure (M2 and M8) and those indicative of neutral evolutionary processes (M1 and M7), as detailed in Anisimova and Yang (2007). All identified sites exhibiting positive selection (Supplementary Tables S2, S3), were exactly mapped onto the sequence alignment. Subsequently, these sites were located on the 3D structure of the protein from the Protein Data Bank (PDB ID: 8ofn). This process enabled us to pinpoint the specific genotypes of YFV that exhibited mutations at these select sites. This mapping not only highlighted the spatial distribution of these mutations on the protein structure but also provided insights into the genotype-specific evolutionary adaptations of YFV.

### 4.10 Deimmunization prediction

The deimmunization prediction analysis we conducted follows the methodology described by Coronado et al. (2019). This process involves using a computational tool as described by Dhanda et al. (2018), which first identifies immunodominant regions in proteins and then predicts amino acid substitutions that could yield non-immunogenic variants of these proteins. Additionally, the tool assesses if these substitutions in immunogenic peptides might alter immunogenic sites in adjacent peptides. Our specific focus was to evaluate the role of a uniquely positively selected site found in the Peruvian/Bolivian/Colombian strain clade of the YFV envelope protein, in contrast to other positively selected sites identified in different YFV genotypes. To this end, we utilized the online deimmunization tool available at IEDB Deimmunization Tool with default settings. For interpreting the outcomes of this analysis, we referred to the scoring guidelines provided on the same platform, which can be accessed at IEDB Deimmunization Help http://tools.iedb.org/deimmunization/help/. This approach allowed us to discern how specific mutations in the envelope protein might affect its immunogenicity, providing valuable insights into potential mechanisms of immune evasion by the virus.

## Data Availability

The authors confirm that the data supporting the findings of this study are available within the article and its Supplementary materials. Raw data including the alignment of all the genomes used and metadata associated are available at https://github.com/LesterJP/YFV_Colombia (Table S5). The YFV sequence obtained from this study is available at Genbank under accession number PP477075. The dataset for this article is available in the NCBI Sequence Read Archive (SRA). It can be accessed from BioProject accession number PRJNA1086472 and NCBI accession: SAMN40377634; SRA: SRS20734849.

## Footnotes

^1^ https://github.com/ViennaRNA/YFVnext
