# Supplementary material for: Emergence, persistence, and positive selection of yellow fever virus in Colombia
Source: Front Microbiol. 2025 Apr 7;16:1548556. doi: 10.3389/fmicb.2025.1548556 (PMC12009951; doi:10.3389/fmicb.2025.1548556)
Supplement: Supplementary file 1 [file Supplementary_file_1.pdf]

Supplementary Information

Emergence, persistence, and positive selection of yellow fever virus in Colombia

Lester J. Perez [1,6Ψ], Laura S. Perez-Restrepo [2,6 Ψ], Karl Ciuoderis [2,6], Jaime Usuga [2,6], Isabel Moreno [2,6], Vanessa Vargas [2,6], Angela J. Arévalo-Arbelaez [2,6], Michael G. Berg [1,6], Gavin A. Cloherty [1,6], Juan Pablo Hernández-Ortiz [2,3], Jorge E. Osorio [2,4, 5, 6]

Table S1. Clinical lab results included as aadditional case findings (reference normal values)

**Laboratory findings at the time of enrollment**	**Hematology**: Hematocrit 43.9% (38.3 to 48.6%); Hemoglobin 14.6 gr/dL (132 to 166 gr/L); Leucocytes 2220/mm3 (3.4 to 9.6 billion cells/L); Neutrophils 68.1%; Lymphocytes 24.9%; Eosinophils 2.4%; Basophils 2.7%, Monocytes 1.9%; Platelet 125000/mm3
	**Urine analysis**: Cloudy, Density 1015; pH 5, Protein 30; Hemoglobin ++; Bacteria
	**Blood smear stain**: Negative for Hemoparasites
	**Blood chemistry**: ASA 4450 U/L (10–40 U/L); ALT 2050 U/L (10–40 U/L); Serum Creatinine 1.12 mg/dL (0.7 to 1.3 mg/dL); Serum Protein C reactive: 4 mg/L (0.8-1.0 mg/dL)
	**Abdominal Echography**: Liver, Pancreas, Spleen and Kidney enlarged.
**Preliminary Diagnosis**	Pyelonephritis based on urinalysis. Urinary tract infection; Probable Dengue with alarm sign (Abdominal pain). Initial support treatment with antipyretic, antiemetic, fluids-Sodium Chloride.
**Actions taken by medical staff**	Patient kept under observation due to the alarm signs
**Clinical evolution one day after enrollment**	Jaundice, persistent abdominal pain on the left side.
**Laboratory findings one day after enrollment**	ASA: 3260 U/L(10–40 U/L) **-increased;** ALT: 2280 U/L (10–40 U/L) **-increased**. Total Bilirubin: 2.67 mg/dL (0.3–1.0 mg/dL) **-increased**; Direct Bilirubin: 2 mg/dL (0.1–0.3 mg/dL) **-increased**
**Lab testing for blood-borne pathogens**	Hepatitis B surface Antigen test Negative; Hepatitis C Antibody test Negative; HIV Antibody test Negative; Leptospira PCR test Negative

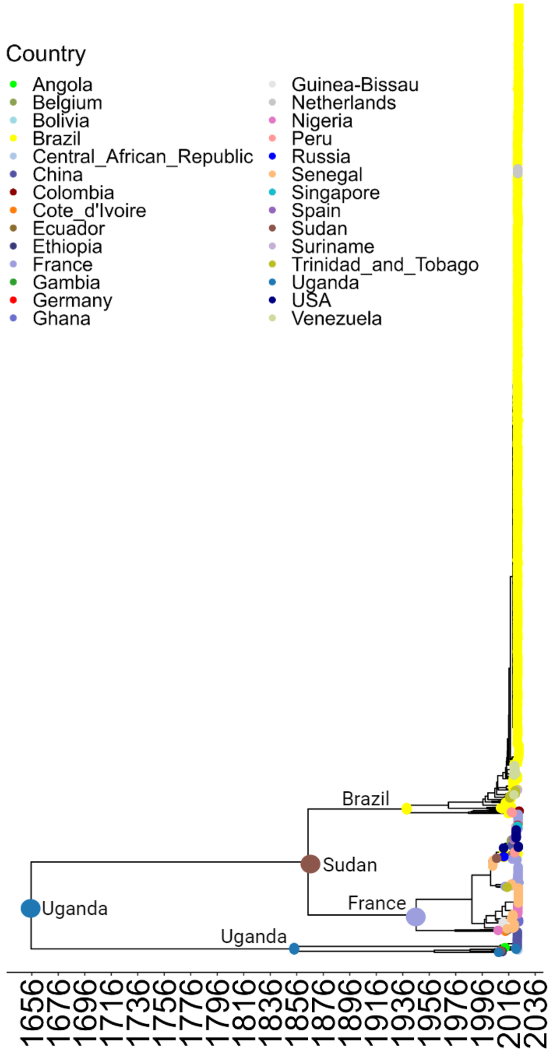

Figure S1. Discrete Phylogeographic Analysis of YFV. Summarized Maximum Clade Credibility (MCC) tree, identifying Uganda as the most probable origin of the YFV and its further proliferation.

Table S2. PAML branch-site model A analysis to identify branches under episodic positive selection in SamII genotype.

**Foreground branches**	**Parameters$^{n.m}$**	**-lnL$^{n.m}$**	**Parameters$^{a.m}$**	**-lnL$^{a.m}$**	**-2ΔlnL**	**Positively selected sites**
Clade:<br>*MF004382/Bolivia/1999*<br>*KF907504/Bolivia/1999*<br>*LET1450/Colombia/2023*	P_0= 0. 97871<br>P_1= 0. 00882<br>P_{2a}= 0. 01236<br>P_{2b}= 0. 00011<br>ω_o= 0. 02042<br>ω_1= 1.0000<br>ω_2=1.0000	19210.250471	P_0= 0. 98633<br>P_1= 0. 00849<br>P_{2a}= 0. 00514<br>P_{2b}= 0. 00004<br>ω_o= 0. 02134<br>ω_1=1.0000<br>ω_2= **2.50760**	19214.061652	**7.62****	**T1185V**

lnL: log-likelihood scores;
n.m: null model; a.m: alternative model
*$p<0.05$, χ^2= 3.84; **$p<0.01$, χ^2= 5.99

Table S3. Positively selected sites and parameters estimated by the CODEML program implemented in the PAML package.

Gene	Model	log-likelihood score						Positive site
Envelope	M1	-3238.261710	p0= 0.61484	p1= 0.38516				-
	M2	-3164.931797	ω_2= 15.15026	p0= 0.44789	p1= 0.50734	p2= 0.04477		70, 140, 155, 177, 200, 227, 243, 268, 305, 331, 343, 344
	M7	-3238.320194	p= 0.00506	q= 0.00758				-
	M8	-3165.013813	ω_2= 14.57338	p0= 0.95534	p= 0.00502	q= 0.00500	(p1= 0.04466)	70, 140, 155, 177, 200, 227, 243, 268, 305, 331, 343, 344

codons selected with a posterior probability greater than 0.99 belonging to the positively selected class ($\omega > 1$)

Table S4. Statistical analysis to avoid false estimations of sites under positive pressure selection.

Gene	Models compared	-2Δl	d.f	dN/dS
Envelope	M1 vs M2	146.65**	2	15.15
	M7 vs M8	146.61**	2	14.57

Neutral models (M1 and M7) were compared to selection models (M2 and M8)
*, $P < 0.05$ **, $P < 0.01$ ($\chi^2_{0.05,2}$= 5.99, $\chi^2_{0.01,2}$= 9.21).
Δl: likelihood-ratio statistic; d.f., degrees of freedom between nested models.
